# Trabecular and cortical mandibular bone investigation in familial adenomatous polyposis patients

**DOI:** 10.1038/s41598-021-88513-z

**Published:** 2021-04-28

**Authors:** Camila Pacheco-Pereira, Yuri Silvestre-Barbosa, Fabiana T. Almeida, Hassem Geha, Andre F. Leite, Eliete N. S. Guerra

**Affiliations:** 1 Faculty of Medicine and Dentistry, School of Dentistry, University of Alberta, Alberta, Canada; 2 Department of Comprehensive Dentistry, School of Dentistry, University of Texas Health,, San Antonio, USA; 3 Health Sciences Faculty, University of Brasília, Brasília, Brazil

**Keywords:** Anatomy, Diseases, Oncology, Signs and symptoms

## Abstract

Mandibular cortical and trabecular bone abnormalities in patients with familial adenomatous polyposis (FAP) were evaluated using dental panoramic radiographs (DPR) radiomorphometric indices and fractal dimension (FD). Sixty DPRs from 15 FAP patients and 45 healthy controls were evaluated. FAP group was composed of 33.3% females and 66.6% males, age_mean_ = 37.2 years (SD 15.79). The non-FAP group was paired by gender and sex. The parameters analyzed were: FD of the trabecular bone in four regions of interest (ROI), mandibular cortical index (MCI) and width (MCW). FD values were lower for the FAP group. Statistically significance differences were shown by ROI 2 and 3 anteriorly to the mental foramen bilaterally, p = 0.001, and p = 0.006. The ROI 1 and 4, at the mandibular angle trabeculae, indicated statistical significances on the right side (p = 0.036) and no differences on the left side (p = 0.091). There was no significant difference in MCI and MCW when the groups were compared, MCW (L) p = 0.247, and MCW (R) p = 0.070. Fractal values of FAP patients' mandibular trabecular bone were lower than healthy controls. The radiomorphometric indices MCI and MCW were not useful for analyzing the cortical bone pattern. Therefore, FD is a promising tool for detection of abnormal bone structure in DPRs and for supporting the appropriate referral of FAP patients.

## Introduction

Colorectal cancer (CRC) is the third cause of cancer-associated death worldwide, and responsible for almost 9% of all deaths^1^. FAP is an autosomal dominant disorder caused by mutations in the Adenomatous Polyposis Coli gene (*APC*)^2,3^. Its prevalence is estimated in 1 case for 6800–29,000 people in whom 1% of CRC cases are caused by *APC* mutation^1^. Clinically, nearly 100% of adult FAP patients develop multiple colorectal adenomas; in most cases, by the fourth decade of life leading to a deteriorated quality of life and survival rate^2,4,5^. It is estimated that 11–25% of the FAP cases will occur for the first time due to de novo mutations, without a family history^6^.

Besides the typical colorectal alterations, FAP patients present several well-known extraintestinal manifestations^2,3,7^. Amongst these, the literature describes classic dental and osseous alterations of the jaws^7,8^. A systematic review revealed the frequency of focal areas of sclerotic bone/osteosclerosis (65.3%) was meaningful^5^. Interestingly, studies have been demonstrated that FAP patients with heterozygous *APC* mutations may present increased bone mineral density (BMD)^3,9^. The role of the *APC* mutation in the alteration of the bone turnover is through β-catenin regulation and activation of the Wnt-protein pathway resulting in increased bone deposition^10^.

Maxillomandibular complex bone pattern changes are commonly detected during routine dental examinations. The dental panoramic radiograph (DPR) is one of the most common extraoral dental imaging modalities. Besides, the DPR is considered a very useful tool to opportunistically identify systemic disorders^11^. Although several studies are evaluating osseous alterations detected via DPR, this mostly observed different results of low BMD in a systemic condition such as osteoporosis^12^. Given that, authors have established radiomorphometric indices and fractal dimension (FD) analysis for assessment of the cortical and trabecular bone patterns using dental imaging^13–16^. The MCW and the morphology of the inferior cortex of the mandible are considered the best predictors of BMD in panoramic radiographs^16^. Besides, the FD is the most commonly used mathematical tile-counting method to assess the bone architecture^17^, and previous studies established the bone texture evaluation of patients with multiple conditions^11,12,18^. Some previous studies assumed that the trabecular bone shows fractal characteristics, self-similarity and the lack of a well-defined range due to a branched structure^19–21^. The method is a promising and cost-effective tool claiming to be a predictor of abnormalities in the complex trabecular bone architecture^22^.

The trabecular bone pattern of FAP patients is affected by osseous manifestations. The possibility of early detection of the disease by DPR by dentists in a clinical practice should be emphasized^8^. It is accepted that the high incidence of sclerotic osseous lesions in the maxillomandibular complex of FAP patients is related to the aforementioned mechanisms while *APC* mutation slightly increases BMD^3^. Although this skeletal effect may occur in FAP patients, the tumour burden is a key factor underlying BMD decline and bone fragility in CRC patients. Therefore, the positive effect of *APC* mutation on bones might be abrogated by elevated intestinal tumour burden. Thus, patients with CRC may even present decreased BMD and elevated fracture risk^23^. To our knowledge, there is no available data on the pattern of the mandibular bone in FAP patients showing the influence of this condition on the mandibular bone turnover. Therefore, we aim to evaluate the mandibular cortices and trabecular bone structure in subjects with FAP and matched controls through DPR radiomorphometric indices and FD.

## Materials and methods

The Ethics Committee of the Health Sciences Faculty, University of Brasilia approved the study (protocol number 493.502), and written informed consent was obtained from all subjects. This matched observational study was carried out following the Declaration of Helsinki and planned to analyze the mandibular bone structure of FAP patients through dental radiographs.

### Study population: familial adenomatous polyposis and a matched-control group

All the available pool of patients referred by the Division of Coloproctology University Hospital of Brasilia that performed DPRs at the Oral Care Center for Inherited Diseases, University Hospital of Brasilia was assessed. From the 60 investigated DPRs, 15 were from FAP patients and 45 matched controls. FAP group was composed of 33.3% females (n = 5) and 66.7% males (n = 10), with a mean age of 37.2 years (SD 15.79). The non-FAP group had 15 females and 30 males, with a mean age of 38.3 years (SD 15.55), *p* = 1.000.

Group 1 was composed of DPRs of FAP patients diagnosed by the standard of care, with no associated metabolic diseases. Patients in this group had a clinical diagnosis of classic FAP which is characterized by the identification of multiple (> 100) colorectal adenomatous polyps on the colonoscopy exam. From these, five patients had a CRC diagnosis but were not in treatment. Some patients in our sample had previously been identified with a heterozygous mutation in the *APC* gene. The genetic characterization was recently detailed by Almeida et al.^24^, and genetic data from the remaining patients of this sample with *APC* causative variant have not been published yet. Group 2 was composed of non-FAP individuals; with no risk of FAP due to familial expression, absence of metabolic bone diseases or a declared developmental and genetic disease. These patients were otherwise healthy with a noncontributory medical history.

Due to the nature of this rare disease, a convenience sample from a center of care of FAP patients were adopted. Groups 1 and 2 were matched by nationality, gender, and age on a proportion of one FAP to three healthy controls; both groups were composed of dentate individuals. Regarding the number of teeth, there was no significant difference between FAP patients (median = 26 teeth, 6–32 teeth) and the control group (median = 28 teeth, 11–32 teeth), *p* = 0.13 (Supplementary Table S1). DPRs were excluded from the sample whether these resulted from 3D technique reconstructions or presenting inadequate positioning during acquisition that could compromise the evaluation of findings. Patients with previous orthognathic surgeries or history of any fracture in the maxillomandibular complex were not accepted. As well, inadequate radiographs with ghost image superimpositions or local bone reactions that could alter the areas of interest or interfere in the evaluation were also excluded.

Clinical characteristics from 10 FAP patients were described in a previous study^24^. The newest recruited FAP patients (n = 5) received a paper-pamphlet containing all the information regarding the study and the informed consent to be signed. They had access to the research project team for further contact if more clarification was needed. The selected DPRs were taken in one of these three machines, the analogic Rotograph Plus (Villa Sistemi Medicalli, Buccinasco, Milan, Italy) and the digital systems from Kodak 8000C (Digital Panoramic, Trophy, France) and Orthophos CD (TS, Dentsply Sirona, Germany). The patients were positioned for acquisition following a standardized protocol in which the vertical orientation line was aligned with the patient mid-sagittal plane and the horizontal one based on the Frankfort plane parallel to the floor; the technicians were calibrated by the same institution and Oral and maxillofacial radiologist (OMR).

For training purposes, an OMR (Evaluator 1) and an Oral and Maxillofacial Surgery resident experienced in FD (Evaluator 2) did the measurements independently using the same computer (Lenovo 15.6″ Intel Core i5-7200U 8 GB RAM 1 TB HDD Windows 10 LED HD resolution 1366 × 768) at two different times, within a 1-week washout interval following a computer-generated randomized list of 10 DPRs. Both observers were blinded to the diagnosis of FAP, risk, or normality status of patients. One evaluator made the final FD and the mandibular cortical measurements in all 60 DPRs.

### The fractal dimension analysis

**Figure 1:**
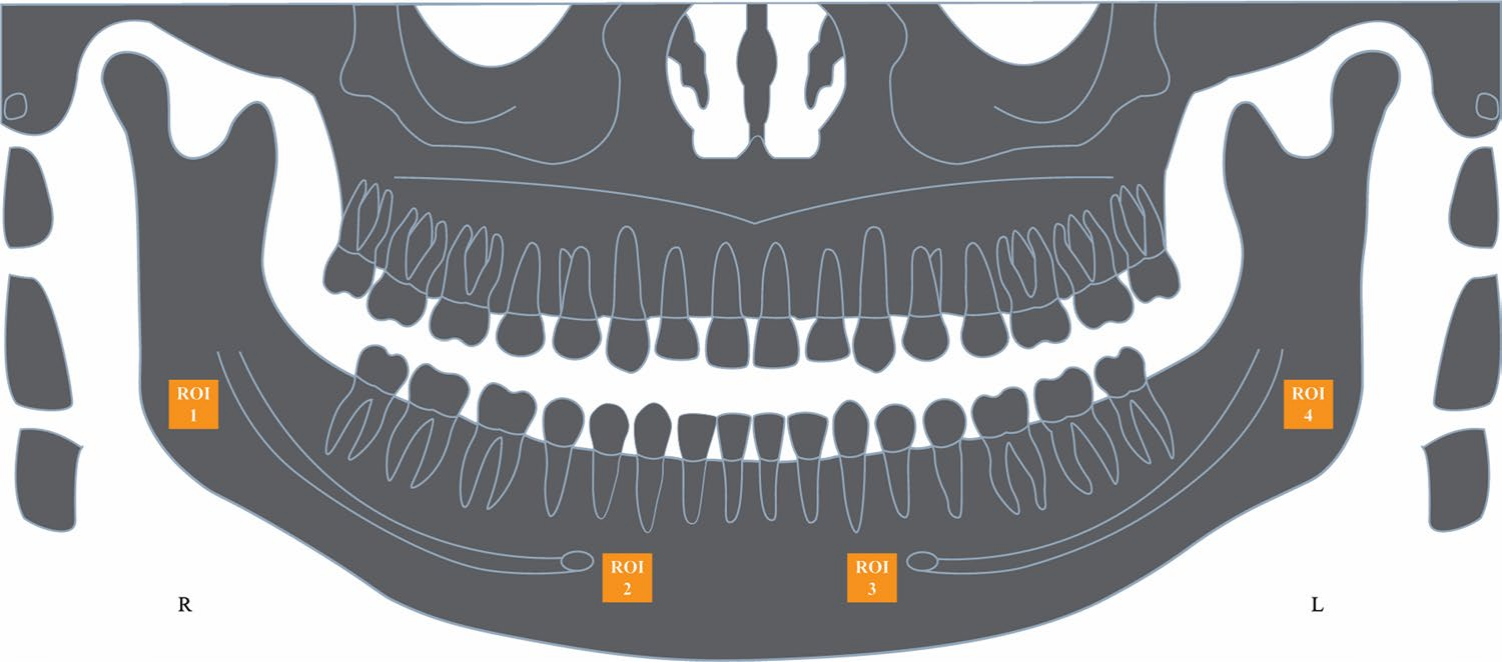
Schema on the elected regions of interest (ROI). *ROI 1* right mandibular angle, *ROI 2* in the trabecular bone, 2 mm anterior to right mental foramen, *ROI 3* in the trabecular bone, 2 mm anterior to left mental foramen, *ROI 4* left mandibular angle.

Trabecular bone areas designated as Regions of Interest (ROI) were chosen for the FD analysis, as per Fig. 1. These were standardized as a square of 100 × 100-pixels and were selected based on previously validated methods as follows. ROI 1: the area above the mandibular angle, below the right mandibular canal and posterior to the molar region, to avoid interference of the masticatory stress in the trabecular bone^18,25,26^. ROI 2: in the trabecular bone, 2 mm anterior to right mental foramen^15,27^. ROI 3: in the trabecular bone, 2 mm anterior to left mental foramen^15,28^. ROI 4: the area above the left mandibular angle, below the mandibular canal and posterior to the molar region^18,25^. These areas were cautiously selected to avoid overlapping of anatomical landmarks such as mandibular canal and cortical bone, and usual radiographic findings of FAP patients such as osteomas, idiopathic osteosclerosis, and supernumeraries.

**Figure 2:**
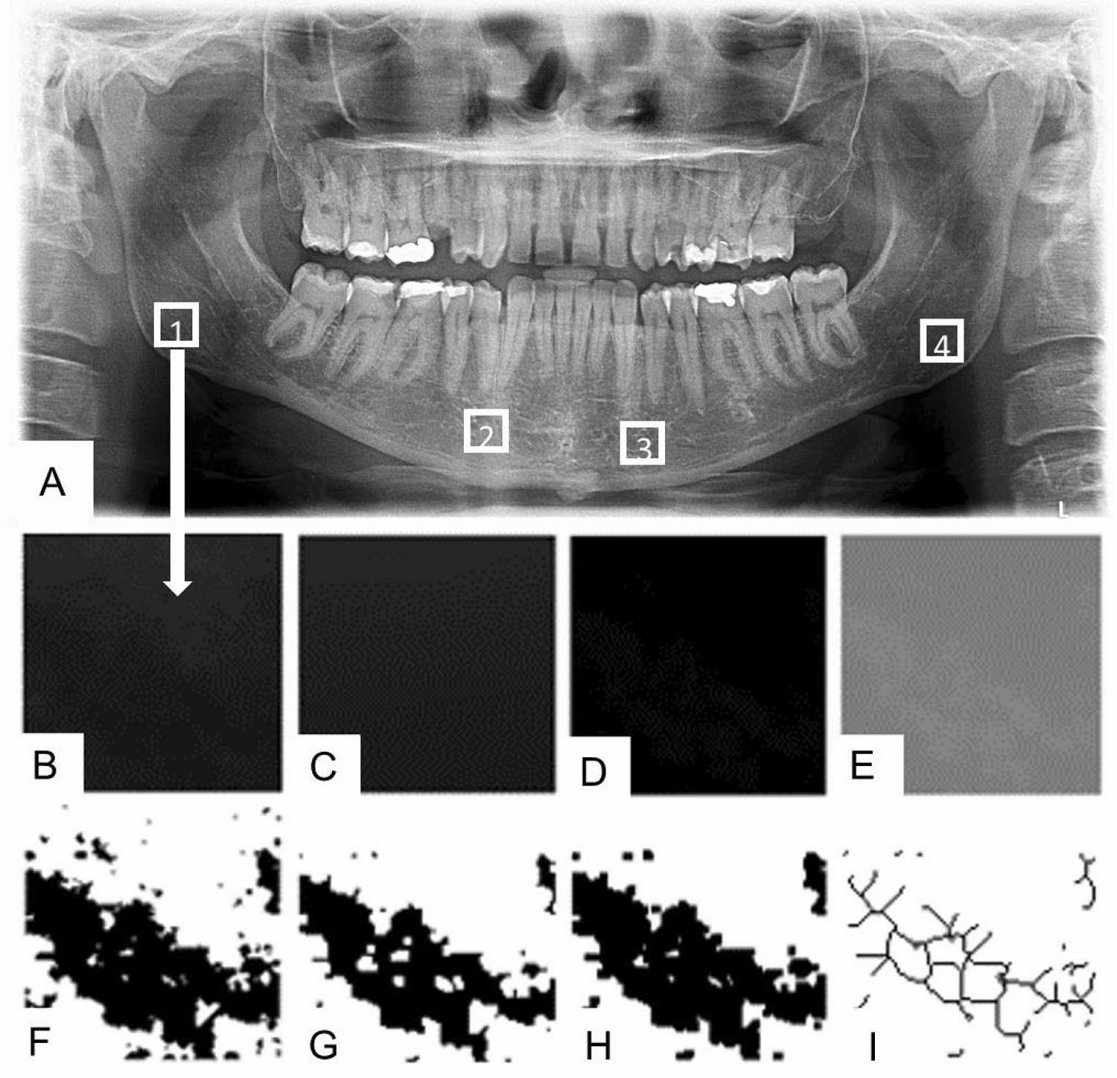
Fractal dimension analysis sequence adopted by this study. FD adopted methodology. The ROIs were standardized as a square of 100 × 100 pixels, cropped and duplicated (**A**). The duplicated image was blurred with a gaussian filter (sigma, 35) to remove large-scale variations in brightness on the image (**B**). The blurred image was subtracted from the original ROI image and a gray value of 128 was added at each pixel location (**C**). The resultant image was made binary and, within this process, the regions that represent trabecular bone were set to black and marrow spaces were set to white (**D**). The image was eroded and dilated to reduce the noise (**E**). After dilation, the image was skeletonized (**F**), and the FD analysis pursuit (**G**). The FD was calculated by the box-counting method (**H**); the widths of these square boxes were 2, 3, 4, 6, 8, 12, 16, 32 and 64 pixels (**I**)*.*

All digital DPRs (n = 11) were stored with a matrix of 7008 × 2975 pixels; the analogic ones (n = 46) were digitalized using the same scanner (Epson 1680 Pro; Seiko Epson Corporation, Japan), with 8-bit grayscale, 300 dpi resolution and stored using the same parameters. The trabecular bone structure was analyzed using ImageJ 1.52a (US National Institute of Health, public domain software available at http://rsbweb.nih.gov/ij). The images were processed similarly to a methodology validated by previous studies, especially following the methods of studies from the same research group^15,18^ and based on the steps validated by White and Rudolph^29^. Figure 2 details the FD analysis sequence adopted by this study.

### The mandibular cortical index (MCI) assessment

**Figure 3:**
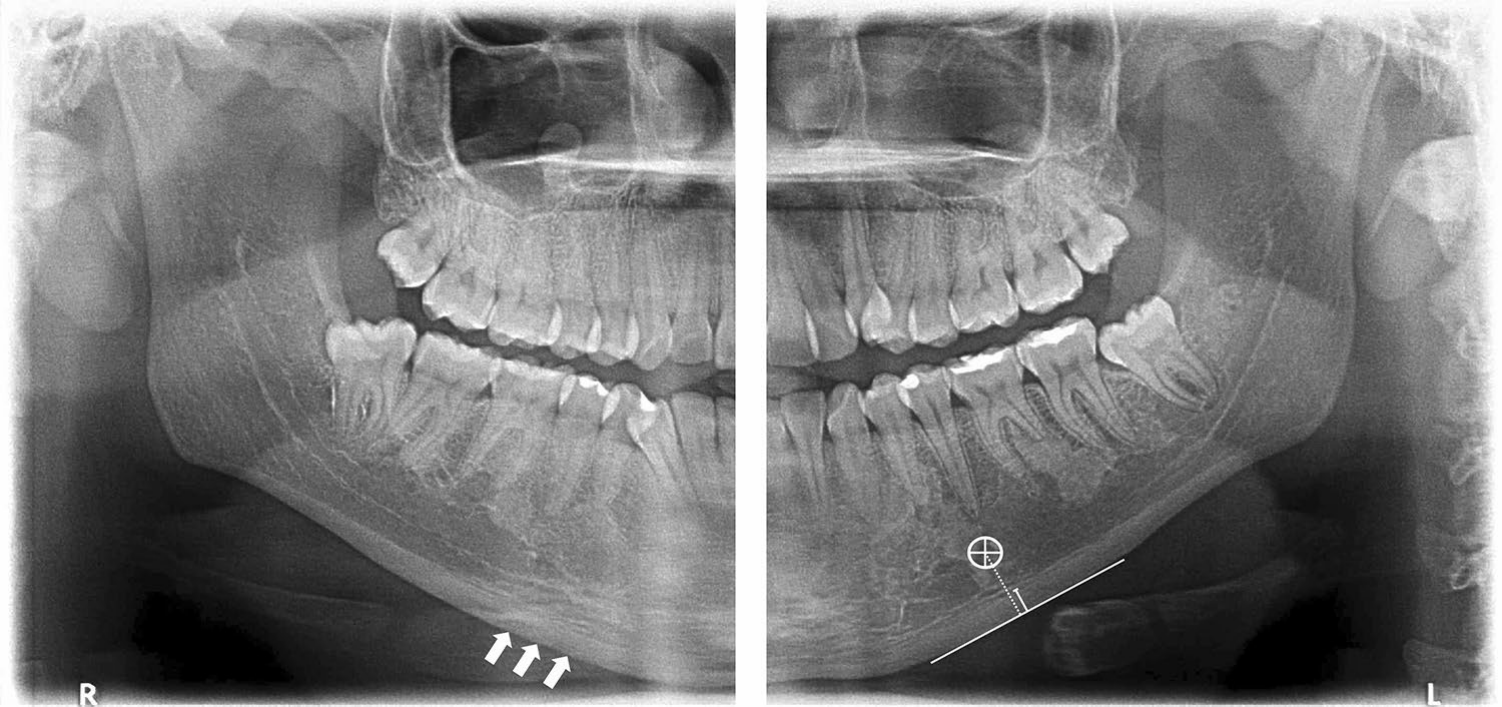
Panoramic radiograph representing the MCI assessment and tracing for MCW measurement. The left cropped DPR shows the inferior cortical border of the mandible that was evaluated qualitatively as C1 = considered homogeneous, C2 = mild erosions and C3 = multiple erosions appearance, clearly porous. The right cropped DPR radiograph showing the mandibular cortical width index. The mental foramen is delimited on the radiograph, and two parallel lines are drawn to demarcate the upper and lower edges of the mandibular cortex. A third line is drawn in the center of the mental foramen and perpendicular to the two cortical lines. The cortical width is determined by measuring the parallel line that surrounds the two structures.

The qualitative assessments of the right and left mandibular cortical bones were made according to the well-established MCI classification validated by Klemetti et al.^30^. The MCI is known for having a very high diagnostic capability when compared to the reference-standard technique for estimation of the BMD, the dual X-ray absorptiometry (DXA). MCI categorizes the appearance of the inferior mandibular cortical thickness as per Fig. 3. Classified as C1: the endosteal margin of the cortex is even and sharp on both sides, C2: the margin shows semilunar defects, lacunar resorption appearance and/or it seems to form endosteal cortical residues in one or both sides, C3: the cortical layer forms heavy endosteal residues. The MCI analysis was performed bilaterally and not allowed to adjust image brightness and contrast to prevent possible interferences.

### The mandibular cortical width (MCW) measurement

The quantitative measurements of the MCW followed Taguchi et al. validated protocols^16^. The ultimate measurement was defined based on the MCW of the mandible body on both sides. The center of the foramen was located, a line extending inferiorly and reaching the lower mandible border in 90° was the point of reference. Support lines were drawn to define the slope and position of the mandibular axis 2D. All indices were measured along this extension line presented in Fig. 3.

### Statistical analysis

A reliability test was applied at two different times; within a 1-week washout interval during training and following the final sample measurements. The Intraclass Correlation Coefficient (ICC) was interpreted by Portney and Watkins guidelines (2009)^31^. Extreme values of the observed variables that were not normally distributed or homoscedastic were checked with Cook’s Distance. The outcomes of the ROI were independently analyzed and left and right sides were assessed. Age, FD measurements, MCI, and MCW were compared between FAP and control groups by parametric Student *t* test. A two-factor ANOVA, factor 1: sex, factor 2: group, was applied to estimate the effects of sex group interactions. The number of teeth was compared between both groups by Mann–Whitney non-parametric test. The Chi-square statistic was applied to verify the distribution of the MCI based on the observed counts and the expected counts if there were no relationship at all in the population. A power analysis presented the actual power achieved for post hoc tests. A *p*-value < 0.05 was considered statistically significant. Statistical Package for Social Sciences (SPSS Statistics 24 software, IBM, Armonk NY) was used for statistical analysis.

### Ethical approval

The Ethics Committee of the Health Sciences Faculty, University of Brasilia approved the study protocol number 493.502.

## Results

### Strong agreement and consistency between evaluators

During training, the intra-rater reliability coefficient was as follows: evaluator 1, ICC = 0.958 (CI = 0.846–0.989) and evaluator 2, ICC = 0.98 (CI = 0.917–0.995). The excellent inter-rater reliability coefficient of the two evaluators is confirmed by an ICC = 0.992 (CI = 0.976–0.998). Also, a Cronbach’s alpha at 0.991 was found when the intra and inter-reliability were considered. For the final sample measurements, the evaluator consistency shows an almost perfect agreement. MCI = 0.967 (CI 0.945–0.980), MCW_(R)_ = 0.995 (0.991–0.997) and MCW_(L)_ = 0.983 (CI 0.972–0.990). For the FD analysis, there was no variation between the first and second fractal values, resulting in an ICC = 100%.

### Fractal dimension values were lower in FAP patients

Table 1 presents the mean FD values for each ROI and the comparison between groups. The mean FD values of FAP were lower than that of the non-FAP in ROI 2 (1.162 SD 0.076, *p* < 0.005), ROI 3 (1.184 SD 0.101, *p* = 0.006) and ROI 4 (1.137 SD 0.089, *p* = 0.036). A more expressive difference was found for ROI 2 and no statistical difference was found in ROI 1 (1.193 SD 0.067, *p* = 0.91).

**Table 1:** Mean fractal dimension on each trabecular area in the FAP and non-FAP groups.

	FAP (n = 15)		Non-FAP (n = 45)		‘t’ value	*p* value
	Mean	± SD	Mean	± SD		
FD-ROI 1	1.193	0.067	1.233	0.082	1.716	0.091; NS
FD-ROI 2	1.162	0.076	1.253	0.067	4.403	< 0.005*
FD-ROI 3	1.184	0.101	1.253	0.075	2.824	0.006*
FD-ROI 4	1.137	0.089	1.206	0.112	2.151	0.036*

NS: *p* > 0.05; *p < 0.05: Significant; ‘t’ = Student t test.

*FD *fractal dimension, *NS *not significant, *ROI *region of interest, *ROI 1* right mandibular angle, *ROI 2* in the trabecular bone, 2 mm anterior to right mental foramen, *ROI 3* in the trabecular bone, 2 mm anterior to left mental foramen, *ROI 4* left mandibular angle, *SD *standard deviation.

A power analysis was conducted based on a post hoc analyses by using G*Power 3^32^. The difference between two independent group means using a two-tailed test, a medium effect size (d = 0.50) and an alpha of 5% significance level was tested. It expressed the actual power achieved in the statistically significant findings of ROI 2, 3 and 4. The effect size was calculated from the mean and standard deviations of these variables. Results showed a calculated power for ROI 2 = 98.7% considered very good, ROI = 72.5% considered acceptable, and a lower power for ROI 4 = 61.4%.

### No difference in the MCI and MCW between groups

The mandibular cortex was associated with an ordinal classification of the morphology of the inferior cortex^33^. Table 2 presents the MCI distribution in two groups, Group I showed 12 FAP cortical qualitatively classified as C1, and 20% of the sample presenting semilunar defects—C2. The matched controls had 75.6% of the mandibular cortices as C1, 20% as C2 and 4.4% as being porous—C3. The MCI was not significant when both groups were compared, *p* = 0.706 and χ^2^ = 0.696. The C1 degree was the most frequent in both groups (*p* = 0.706).

**Table 2:** Distribution of mandibular cortical index (MCI) in the two groups.

MCI	FAP (n = 15)		Non-FAP (n = 45)		χ^2^ value	*p* value
	N	%	N	%		
C1	12	80.0	34	75.6	0.696	0.706; NS
C2	3	20.0	9	20.0		
C3	–	–	2	4.4		
Total	15	100.0	45	100.0		

Results are given as percentages. *p*-values were determined using Chi-Square Test (χ^2^); NS: *p* > 0.05.

*C1* the endosteal margin of the cortex is even and sharp on both sides, *C2* the margin shows semilunar defects, lacunar resorption appearance and/or it seems to form endosteal cortical residues in one or both sides, *C3* the cortical layer forms heavy endosteal residues and it is clearly porous, *MCI *mandibular cortical index, *N *number, *NS *not significant.

For FAP, the mean MCW _right (R)_ = 3.399 (SD 0.506) and MCW _left (L)_ = 3.382 (SD 0.678). The non-FAP showed a mean MCW_(R)_ = 3.368 (SD 0.732) and MCW_(L)_ = 3.718 (SD 0.589). These values show no statistical significance for both sides when the groups were compared, MCW_(L)_ *p* = 0.247 and t = 1.170 and for MCW_(R)_ t = 1.848 and *p* = 0.070, see Table 3.

**Table 3:** Mandibular cortical width in the FAP and non-FAP groups.

	FAP (n = 15)		non-FAP (n = 45)		‘t’ value	*p* value
	Mean	± SD	Mean	± SD		
MCW (R)	3.399 mm	0.506	3.638 mm	0.732	1.170	0.247; NS
MCW (L)	3.382 mm	0.678	3.718 mm	0.589	1.846	0.070; NS

Results are given as mean ± SD. p-values were determined using Student *t* test; NS: *p* > 0.05.

*L *left side, *MCW *mandibular cortical width, *mm *millimeters, *NS *not significant, *R *right side.

Considering the gender of the patients, no significant differences were found for all radiomorphometric indices and fractal values in FAP, *p* > 0.074. In the non-FAP, significant differences between males and females were only found for MCWR (*p* = 0.003) and MCWL (*p* = 0.021) values. When Table 2 is compared to Supplementary Tables S2, S3 and S4, we assume that the variability in FD is higher in the male group influenced by age. Age in male groups presented significantly higher variances when compared to age in female groups. On the other hand, the mean age between males (42.5 years) and females (38.6 years) were not statistically different, *p* > 005. See Supplementary Tables S2, S3, S4, and S5. When considering all individuals, no differences were found between males and females for all variables, except for MCW_right_ and ROI 4 (ANOVA test, *p* = 0.032 and *p* = 0.043, respectively).

## Discussion

Although the extraintestinal manifestations of FAP, specifically the osseous and dental alterations, were addressed previously^3,5,7,8,34^, this study adds unpublished and relevant knowledge while evaluating the mandibular cortical and trabecular radiomorphometric indices of FAP and matched healthy patients in DPRs.

The FD analysis value reflects how much a fractal completes the trabecular bone spaces; it is considered a texture evaluation. Previous authors established that a high FD correlates to a greater bone complexity and a lower FD is indicative of a simpler structure^35^. For the fractal analysis, we have selected the ROI 100 × 100-pixel size (4.23 mm) based on validated fractal studies^36,37^. These studies showed that FD of the mandibular trabecular bone has optimal characterization when the tile size ranges from 0.025 to 4.25 mm. While the BMD is frequently used in studies investigating changes in the bone mass, the FD values are related to the bone texture and the complexity of patterns. It is essential to understand that FD is independent of the BMD, not synonymous. The BMD scores represent the disruption of the bone structure and replacement of the cancellous bone by non-collagenous proteins and monitor bone fragility^38^. In FAP, the structural alterations in bone were investigated in animal studies. As shown by Holmen et al.^10^, *APC* mutated mice developed a bone in which the vast majority of the marrow component is absent; they found that the *APC* mutation was linked to a dramatically increased bone deposition associated with its architecture disturbances. The present results indicate that FD measurements may differentiate FAP patients from individuals considered being healthy and having a normal trabecular bone. The mean fractal values found in our sample confirm the assumption of FAP patients having altered mandibular bone architecture.

The fractal values of FAP patients were generally smaller when compared to controls. Areas anterior to the mental foramen showed significant differences (*p* = 0.001 and *p* = 0.006) when both groups were compared. And, this was emphasized in the power analysis. In agreement with a previous study using a smaller ROI at the same location, this area demonstrated an altered bone structure when comparing osteoporotic and normal patients (*p* = 0.032)^39^. However, fractal values can be contradictory when the same disease is investigated. As an example, another study found no significance on a larger squared ROI (*p* = 0.621) on osteoporosis^15^. This variation could be due to disagreement of the results in their studies and could be due to anatomical variations, discrepancies in the methods, techniques for FD, and/or the differences in selecting the regions to be measured. The second area explored by our study, the trabecular bone at the angle of the mandible bilaterally, showed a difference on the left side (*p* = 0.036) between the two groups. In spite of the lack of statistical significance on the right side, we still consider the angle of the mandible an adequate area to assess the bone structure; this, is in agreement with previous studies^18,25,26^. Furthermore, our study carefully eliminated the areas affected by the radiographic findings and normal anatomical variants to prevent misrepresentation of the mandibular trabecular structure represented by the fractal values^40^. The ROIs explored by this study are considered good predictors of bone texture^12,15,18,25–28^. Once these areas are demarcated, the methodology followed is computerized and carries an error-free calculation. For this reason, the one calibrated-evaluator measured the entire sample twice without differences, the evaluator consistency tends to be perfect, and the double measurement is questionable.

The *APC* suppressor effects in the cancellous bone were described by Miclea et al.^3^. FAP patients’ display of an increased mean BMD may be because the *APC* gene regulates bone density. The increased BMD is possible due to the inhibition of the β-catenin that regulates the pathophysiology of bone formation and disorders^9^. The increased accumulation of bone matrix is a consequence of the activation of the Wnt/β-catenin pathway that promotes osteoblast differentiation, leading to bone mass acquisition. Hence, the normal architecture bone structure is altered, and the trabecular structure becomes less complex, resulting in lower FD of trabecular bone projection texture values, as observed in our analysis. The FAP fractal values were approximately 5% lower than the controls. Studies exploring low BMD also show lower fractal values, the bone pattern disruption rationale is not similar, but the bone texture caused by bone cell disbalance will result in decreased fractal values. Interestingly, CRC and osteoporosis represent two global challenges in public health^1,41^.

In our study, the radiomorphometric indices did not show differences between groups. Besides, the cortical bone dimensional changes were associated with systemic diseases^18,42,43^, and these found MCW values higher than those considered in the normal range (MCW < 4 mm)^12,38^. Considering that the patients in our sample are relatively young (~ 37 years), a distinct scenario could be seen with aging individuals since the bone turnover tends to disbalance^38^. In accordance with our results, experiments in CRC mice showed the integrity of the cortical bone. This could also be due to the bone turnover or a short treatment time causing cancellous bone alterations and not affecting the cortical bone^44^.

Our study also aims to create awareness in both the dental and medical community, so these results could be translated clinically. Despite previous literature on FAP patients^7,8^, the utility of the DPRs may surpass the dental evaluation; the opportunistic evaluation of the already taken DPR is essential for the surveillance of systemic conditions and osseous manifestations^11,45^. The clinical importance of this study for FAP patients is the early detection of osseous disorders, as the lower fractal numbers found in our study showed the expected trabecular texture disruption. These values represent a loss on the bone texture even though radiographically, the findings are resultant from bone accumulation. Once the visual inspection of the trabecular bone is performed, a base-pattern analysis is essential.

Thus, the radiographic findings should be attentively correlated to the medical history, and further investigated if a systemic disorder is suspected. FAP patients’ incidental findings are treatable and present no challenge to the routine dental practice. The key point is the early detection, referral and radiographic follow up. We emphasize the selection criteria for radiographs^46^. DPRs are not recommended specifically for FAP surveillance, but rather that the dentists evaluate radiographs taken for dental purposes for this condition as well. The radiographic alterations could be detected in any patient and, if the condition is suspected, referral to the primary care physician is advised. Confirmed FAP children and adults should undergo a colonoscopy assessment before genetic testing^47^. Preventative measures such as the evaluation of existing DPRs could assist with the early detection of CRC to reduce its incidence by approximately 55% and improve their survival rates^1,48^.

Pointing out the limitations of this study, we should consider that few DPRs were conventional film-based and the majority are digital. To minimize the difference, the conventional ones were digitalized and imported to ImageJ using the same spatial parameters and resolution. Besides, DPRs represent a 2D image of a 3D trabecular structure. A 3D analysis will undoubtedly give more valuable information; however, cone beam computed tomography is not a method routinely used for a radiographic follow for the FAP patients due to the ionizing radiation dose concerns and radiation safety precautions.

Since this is not an investigation of bone mineral density; unfortunately, this data is not available for the FAP patients and controls of our sample. As a future approach, the correlation of FAP patient’s fractal analysis with the BMD through DXA could elucidate and reveal the likelihood or risk of bone fracture or possible fracture protection in FAP patients. As well, the standardization of the ROI pixel size in the mandibular body and fractal analysis parameters could ease the comparison of the results between distinct research projects.

## Summary and conclusion

Routinely taken panoramic radiographs are essential for monitoring and surveillance of FAP. By creating awareness in the dental community, our study could be clinically translated by implementing the evaluation of the mandible trabeculae. Preventive measures reduce colorectal cancer incidence and improve the survival rates of FAP patients.

The analysis of the trabecular bone structures of FAP patients showed that most fractal values of the mandibular trabecular bone were lower than the matched controls. Therefore, the FD is promising as a tool for evaluation of the bone abnormalities in these patients. The radiomorphometric indices MCI and MCW were not useful for the analysis of the cortical bone pattern of patients affected by the FAP.

## Supplementary Information

Supplementary Tables.

## References

[1] Fitzmaurice, C. et al. Global, regional, and national cancer incidence, mortality, years of life lost, years lived with disability, and disability-adjusted life-years for 29 cancer groups, 1990 to 2017: a systematic analysis for the global burden of disease study. *JAMA Oncol*. **5,** 1749–1768 (2019). 10.1001/jamaoncol.2019.2996 PMC6777271 31560378

[2] Half E, Bercovich D, Rozen P (2009). Familial adenomatous polyposis. Orphanet J. Rare Dis..

[3] Miclea RL (2010). APC mutations are associated with increased bone mineral density in patients with familial adenomatous polyposis. J. Bone Miner. Res..

[4] Aihara H, Kumar N, Thompson CC (2014). Diagnosis, surveillance, and treatment strategies for familial adenomatous polyposis: rationale and update. Eur. J. Gastroenterol. Hepatol..

[5] Almeida FT (2016). Oral manifestations in patients with familial adenomatous polyposis: A systematic review and meta-analysis. J. Gastroenterol. Hepatol..

[6] Ripa R, Bisgaard ML, Bülow S, De Nielsen FC (2002). novo mutations in familial adenomatous polyposis (FAP). Eur. J. Hum. Genet..

[7] Gardner EJ (1962). Follow-up study of a family group exhibiting dominant inheritance for a syndrome including intestinal polyps, osteomas, fibromas and epidermal cysts. Am. J. Hum. Genet..

[8] Septer S (2018). Dental anomalies in pediatric patients with familial adenomatous polyposis. Fam. Cancer.

[9] Chew S (2012). Copy number variation of the APC gene is associated with regulation of bone mineral density. Bone.

[10] Holmen SL (2005). Essential role of beta-catenin in postnatal bone acquisition. J. Biol. Chem..

[11] Pachêco-Pereira C (2019). Dental imaging of trabecular bone structure for systemic disorder screening: A systematic review. Oral Dis..

[12] Leite AF, Figueiredo PT, Guia CM, Melo NS, de Paula AP (2010). Correlations between seven panoramic radiomorphometric indices and bone mineral density in postmenopausal women. Oral Surg. Oral Med. Oral Pathol. Oral Radiol. Endod..

[13] Gomes CC, de Rezende Barbosa GL, Bello RP, Bóscolo FN, de Almeida SM (2014). A comparison of the mandibular index on panoramic and cross-sectional images from CBCT exams from osteoporosis risk group. Osteoporos. Int..

[14] Mostafa RA, Arnout EA, Abo El-Fotouh MM (2016). Feasibility of cone beam computed tomography radiomorphometric analysis and fractal dimension in assessment of postmenopausal osteoporosis in correlation with dual X-ray absorptiometry. Dentomaxillifac. Radiol..

[15] Sindeaux R (2014). Fractal dimension and mandibular cortical width in normal and osteoporotic men and women. Maturitas.

[16] Taguchi A (1996). Usefulness of panoramic radiography in the diagnosis of postmenopausal osteoporosis in women. Width and morphology of inferior cortex of the mandible. Dentomaxillofac. Radiol..

[17] Geraets WG, van der Stelt PF (2000). Fractal properties of bone. Dentomaxillofac. Radiol..

[18] Apolinário AC (2016). Dental panoramic indices and fractal dimension measurements in osteogenesis imperfecta children under pamidronate treatment. Dentomaxillofac. Radiol..

[19] Sanchez-Molina D (2013). Fractal dimension and mechanical properties of human cortical bone. Med. Eng. Phys..

[20] Neves FS (2020). Assessment of fractal dimension and panoramic radiomorphometric indices in women with celiac disease. Oral Radiol..

[21] Gumussoy I, Miloglu O, Cankaya E, Bayrakdar IS (2016). Fractal properties of the trabecular pattern of the mandible in chronic renal failure. Dentomaxillofac. Radiol..

[22] Ergün S, Saraçoglu A, Güneri P, Ozpinar B (2009). Application of fractal analysis in hyperparathyroidism. Dentomaxillofac. Radiol..

[23] Saul D, Schilling AF, Kosinsky RL (2018). Intestinal inflammation and tumor burden as determinants for bone fragility in APC-driven tumorigenesis. Inflamm. Bowel Dis..

[24] Almeida FT (2020). Dento-osseous anomalies in patients with familial adenomatous polyposis: A follow-up study. Clin. Oral Investig..

[25] Oliveira ML (2013). Relationship between bone mineral density and trabecular bone pattern in postmenopausal osteoporotic Brazilian women. Clin. Oral Investig..

[26] Roberts MG, Graham J, Devlin H (2013). Image texture in dental panoramic radiographs as a potential biomarker of osteoporosis. IEEE Trans. Biomed. Eng..

[27] Taguchi A, Tanimoto K, Suei Y, Wada T (1995). Tooth loss and mandibular osteopenia. Oral Surg. Oral Med. Oral Pathol. Oral Radiol. Endod..

[28] Kathirvelu D, Anburajan M (2014). Prediction of low bone mass using a combinational approach of cortical and trabecular bone measures from dental panoramic radiographs. Proc. Inst. Mech. Eng. H..

[29] White SC, Rudolph DJ (1999). Alterations of the trabecular pattern of the jaws in patients with osteoporosis. Oral Surg. Oral Med. Oral Pathol. Oral Radiol. Endod..

[30] Klemetti E, Kolmakov S, Kröger H (1994). Pantomography in assessment of the osteoporosis risk group. Scand. J. Dent. Res..

[31] Portney LG, Watkins MP (2009). Foundations of Clinical Research: Applications to Practice.

[32] Faul F, Erdfelder E, Lang AG, Buchner A (2007). G*Power 3: A flexible statistical power analysis program for the social, behavioral, and biomedical sciences. Behav. Res. Methods.

[33] Klemetti E, Kolmakow S (1997). Morphology of the mandibular cortex on panoramic radiographs as an indicator of bone quality. Dentomaxillofac. Radiol..

[34] Thakker N (1995). The dental phenotype in familial adenomatous polyposis: Diagnostic application of a weighted scoring system for changes on dental panoramic radiographs. J. Med. Genet..

[35] Sánchez I, Uzcátegui G (2011). Fractals in dentistry. J. Dent..

[36] Huh KH (2011). Fractal analysis of mandibular trabecular bone: Optimal tile sizes for the tile counting method. Imaging Sci. Dent..

[37] Parkinson IH, Fazzalari NL (2000). Methodological principles for fractal analysis of trabecular bone. J. Microsc..

[38] Demontiero O, Vidal C, Duque G (2012). Aging and bone loss: New insights for the clinician. Ther. Adv. Musculoskelet. Dis..

[39] Khojastehpour L, Mogharrabi S, Dabbaghmanesh MH, Iraji Nasrabadi N (2013). Comparison of the mandibular bone densitometry measurement between normal, osteopenic and osteoporotic postmenopausal women. J. Dent. (Tehran).

[40] Chappard C (2005). Anisotropy changes in post-menopausal osteoporosis: Characterization by a new index applied to trabecular bone radiographic images. Osteoporos. Int..

[41] Unnanuntana A, Rebolledo BJ, Khair MM, DiCarlo EF, Lane JM (2011). Diseases affecting bone quality: Beyond osteoporosis. Clin. Orthop. Relat. Res..

[42] Iwata E (2017). Meaning and limitation of cortical bone width measurement with DentaScan in medication-related osteonecrosis of the jaws. Kobe J. Med. Sci..

[43] Kurşun-Çakmak EŞ, Bayrak S (2018). Comparison of fractal dimension analysis and panoramic-based radiomorphometric indices in the assessment of mandibular bone changes in patients with type 1 and type 2 diabetes mellitus. Oral Surg. Oral Med. Oral Pathol. Oral Radiol..

[44] Hamdani G (2008). Dextran sodium sulfate-induced colitis causes rapid bone loss in mice. Bone.

[45] Kato CN (2020). Mandibular radiomorphometric parameters of women with cemento-osseous dysplasia. Dentomaxillofac. Radiol..

[46] American Dental Association Council on Scientific Affairs (2006). The use of dental radiographs: Update and recommendations. J. Am. Dent. Assoc..

[47] Herzig D (2017). The American Society of Colon and Rectal Surgeons Clinical Practice Guidelines for the management of inherited polyposis syndromes. Dis. Colon Rectum.

[48] Heiskanen I, Luostarinen T, Järvinen HJ (2000). Impact of screening examinations on survival in familial adenomatous polyposis. Scand. J. Gastroenterol..

