# Supplementary material for: Trabecular and cortical mandibular bone investigation in familial adenomatous polyposis patients
Source: Sci Rep. 2021 Apr 28;11:9143. doi: 10.1038/s41598-021-88513-z (PMC8080795; doi:10.1038/s41598-021-88513-z)
Supplement: Supplementary file 1 — Supplementary Tables. [file 41598_2021_88513_MOESM1_ESM.doc]

**Trabecular and cortical mandibular bone structure in**

**Familial Adenomatous Polyposis patients**

**Dr. Camila Pacheco-Pereira** – Clinical Assistant Professor at School of Dentistry, Faculty of Medicine and Dentistry, School of Dentistry, University of Alberta, Canada and University of Texas health Sciences Center at San Antonio, Texas, United States. Email: cppereir@ualberta.ca

**Dr. Yuri Silvestre-Barbosa** – Resident, Department of Oral and Maxillofacial Surgery, Health Sciences Faculty, University of Brasília, Brasília, Brazil. Email: [yurisilver5@gmail.com](mailto:yurisilver5@gmail.com)

**Dr. Fabiana T. Almeida** – Clinical Assistant Professor at the Faculty of Medicine and Dentistry, School of Dentistry, University of Alberta, Canada. Email: fabiana@ualberta.ca

**Dr. Hassem Geha** – Associate Professor, Department of Comprehensive Dentistry, School of Dentistry, University of Texas Health – San Antonio, USA. Email: geha@uthscsa.edu

**Dr. Andre F. Leite** – Associate Professor at the Health Sciences Faculty, University of Brasília, Brasília, Brazil. Email: andreleite@unb.br

***Dr. Eliete N. S. Guerra** – Professor at the Health Sciences Faculty, Laboratory of Oral Histopathology, University of Brasília, Brasília, Brazil. Email: elieteneves.unb@gmail.com.br

Supplementary material

**Table S1 –** Teeth Number of Individuals in the FAP and non-FAP groups

|  | Number of teeth | | Median CI | | *p* value |
| --- | --- | --- | --- | --- | --- |
|  | Lowest | Highest |  | 95% |  |
| FAP (n=15) | 6 | 32 | 26 | 18.3 to 30.2 |  |
| Non-FAP (n=45) | 11 | 31 | 28 | 27.0 to 29.3 | 0.13; NS |

P-values were determined using Mann-Witney test; NS: *p* > 0.05. CI=Confidence interval; NS=Not Significant.

**Table S2**: Comparing Mean MCW, MCW and FD-ROI between Males and Females in the FAP group

|  | Males (n = 5) | | Females (n = 10) | | ‘t’ value | *p* value |
| --- | --- | --- | --- | --- | --- | --- |
|  | Mean | ± SD | Mean | ± SD |  |  |
| MCW _(R)_ | 3.540 | 0.587 | 3.329 | 0.478 | 0.749 | 0.467; NS |
| MCW _(L)_ | 3.404 | 0.546 | 3.371 | 0.759 | 0.086 | 0.933; NS |
| FD-ROI 1 | 1.209 | 0.095 | 1.184 | 0.053 | 0.675 | 0.511; NS |
| FD-ROI 2 | 1.131 | 0.094 | 1.178 | 0.065 | 1.138 | 0.276; NS |
| FD-ROI 3 | 1.214 | 0.094 | 1.169 | 0.106 | 0.801 | 0.438; NS |
| FD-ROI 4 | 1.079 | 0.086 | 1.166 | 0.080 | 1.944 | 0.074; NS |

“t” = Student t test; NS: *p* > 0.05.

CI=Confidence Interval; FD=Fractal Dimension; L=Left Side; MCI=Mandibular Cortical Index; MCW=Mandibular Cortical

Width; NS=Not Significant.; R= Right Side; ROI=Region of Interest; ROI 1= Right mandibular angle; ROI 2= In the trabecular

bone, 2mm anterior to right mental foramen; ROI 3= In the trabecular bone, 2mm anterior to left mental foramen; ROI 4= Left

mandibular angle; SD=Standard Deviation.

**Table S3**: Comparing Mean MCW, MCW and FD-ROI between Males and Females in non-FAP group

|  | Males (n = 15) | | Females (n = 30) | | ‘t’ value | *p* value |
| --- | --- | --- | --- | --- | --- | --- |
|  | Mean | ± SD | Mean | ± SD |  |  |
| MCW _(R)_ | 4.085 | 0.559 | 3.415 | 0.712 | 3.181 | 0.003* |
| MCW _(L)_ | 4.001 | 0.446 | 3.577 | 0.608 | 2.395 | 0.021* |
| FD-ROI 1 | 1.219 | 0.051 | 1.239 | 0.093 | 0.785 | 0.437; NS |
| FD-ROI 2 | 1.257 | 0.051 | 1.251 | 0.074 | 0.260 | 0.796; NS |
| FD-ROI 3 | 1.261 | 0.052 | 1.249 | 0.085 | 0.477 | 0.636; NS |
| FD-ROI 4 | 1.239 | 0.092 | 1.189 | 0.119 | 1.437 | 0.158; NS |

“t” = Student t test; NS: *p* > 0.05. *p<0.05; Significant

CI=Confidence Interval; FD=Fractal Dimension; L=Left Side; MCI=Mandibular Cortical Index; MCW=Mandibular Cortical

Width; NS=Not Significant.; R= Right Side; ROI=Region of Interest; ROI 1= Right mandibular angle; ROI 2= In the trabecular

bone, 2mm anterior to right mental foramen; ROI 3= In the trabecular bone, 2mm anterior to left mental foramen; ROI 4= Left

mandibular angle; SD=Standard Deviation.

**Table S4**: Comparing Mean MCW, MCW and FD-ROI between FAP and non-FAP Males

|  | FAP (n = 5) | | Non-FAP (n = 15) | | ‘t’ value | *p* value |
| --- | --- | --- | --- | --- | --- | --- |
|  | Mean | ± SD | Mean | ± SD |  |  |
| MCW _(R)_ | 3.540 | 0.587 | 4.085 | 0.559 | 1.864 | 0.079; NS |
| MCW _(L)_ | 3.404 | 0.546 | 4.001 | 0.446 | 2.462 | 0.024* |
| FD-ROI 1 | 1.209 | 0.095 | 1.219 | 0.051 | 0.292 | 0.774; NS |
| FD-ROI 2 | 1.131 | 0.094 | 1.257 | 0.051 | 3.842 | 0.001* |
| FD-ROI 3 | 1.214 | 0.094 | 1.261 | 0.052 | 1.421 | 0.172; NS |
| FD-ROI 4 | 1.079 | 0.086 | 1.239 | 0.092 | 3.449 | 0.003* |

“t” = Student t test; NS: *p* > 0.05. *p<0.05; Significant

CI=Confidence Interval; FD=Fractal Dimension; L=Left Side; MCI=Mandibular Cortical Index; MCW=Mandibular Cortical

Width; NS=Not Significant.; R= Right Side; ROI=Region of Interest; ROI 1= Right mandibular angle; ROI 2= In the trabecular

bone, 2mm anterior to right mental foramen; ROI 3= In the trabecular bone, 2mm anterior to left mental foramen; ROI 4= Left

mandibular angle; SD=Standard Deviation.

**Table S5**: Comparing Mean MCW, MCW and FD-ROI between FAP and non-FAP Females

|  | FAP (n = 10) | | Non-FAP (n = 30) | | ‘t’ value | *p* value |
| --- | --- | --- | --- | --- | --- | --- |
|  | Mean | ± SD | Mean | ± SD |  |  |
| MCW _(R)_ | 3.329 | 0.478 | 3.415 | 0.712 | 0.353 | 0.726; NS |
| MCW _(L)_ | 3.371 | 0.759 | 3.577 | 0.608 | 0.872 | 0.389; NS |
| FD-ROI 1 | 1.184 | 0.053 | 1.239 | 0.093 | 1.777 | 0.084; NS |
| FD-ROI 2 | 1.178 | 0.065 | 1.251 | 0.074 | 2.786 | 0.008* |
| FD-ROI 3 | 1.169 | 0.106 | 1.249 | 0.085 | 2.435 | 0.020* |
| FD-ROI 4 | 1.166 | 0.080 | 1.189 | 0.119 | 0.564 | 0.576; NS |

“t” = Student t test; NS: *p* > 0.05. *p<0.05; Significant

CI=Confidence Interval; FD=Fractal Dimension; L=Left Side; MCI=Mandibular Cortical Index; MCW=Mandibular Cortical

Width; NS=Not Significant.; R= Right Side; ROI=Region of Interest; ROI 1= Right mandibular angle; ROI 2= In the trabecular

bone, 2mm anterior to right mental foramen; ROI 3= In the trabecular bone, 2mm anterior to left mental foramen; ROI 4= Left

mandibular angle; SD=Standard Deviation.
